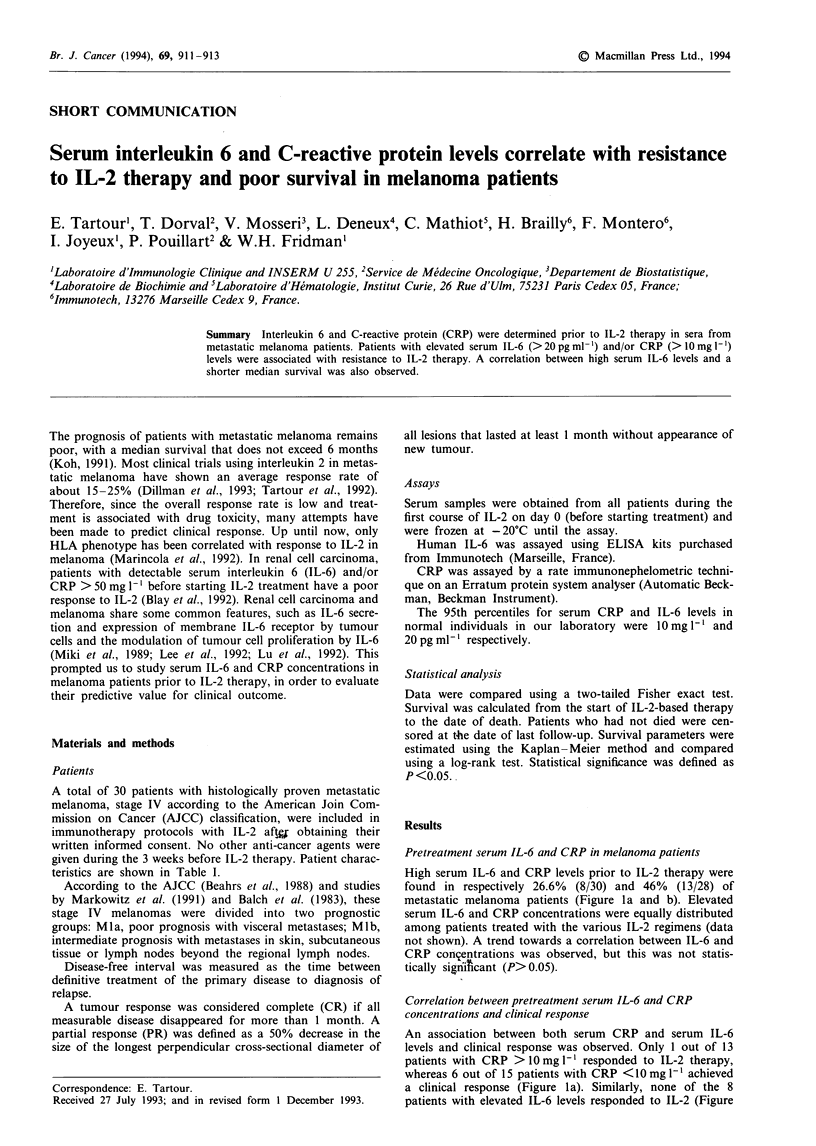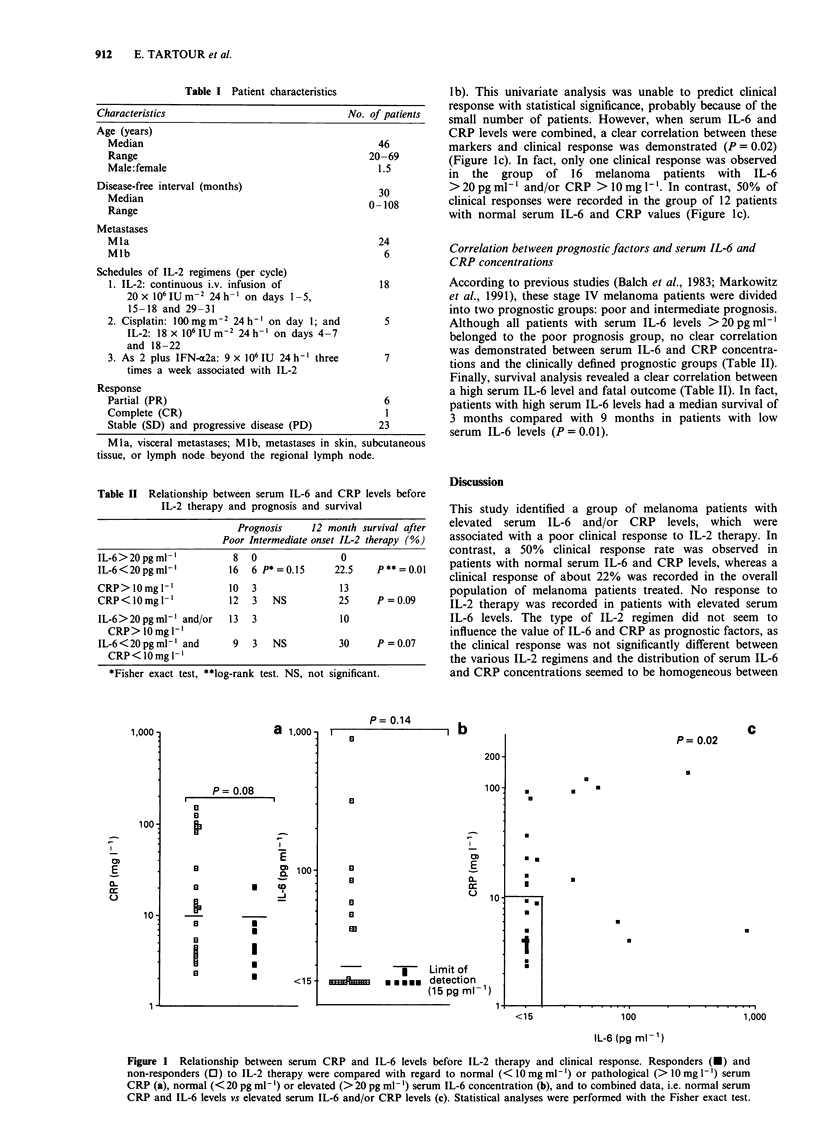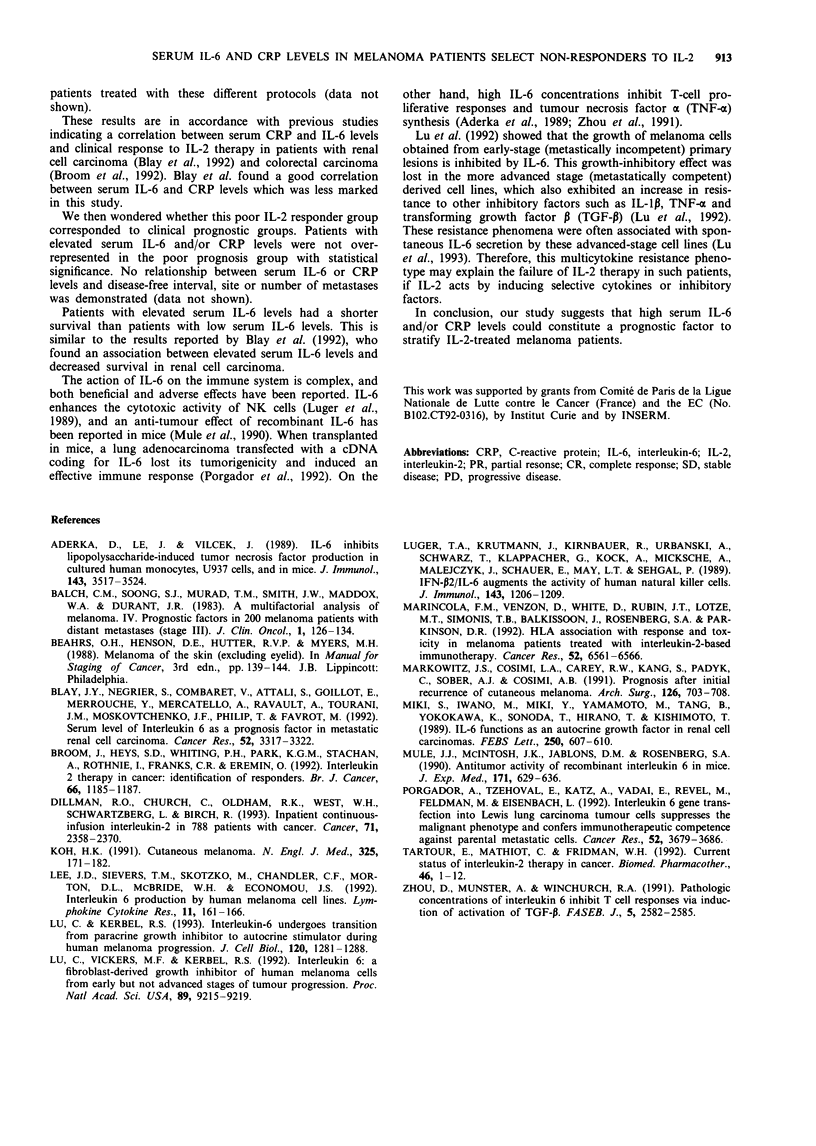# Serum interleukin 6 and C-reactive protein levels correlate with resistance to IL-2 therapy and poor survival in melanoma patients.

**DOI:** 10.1038/bjc.1994.175

**Published:** 1994-05

**Authors:** E. Tartour, T. Dorval, V. Mosseri, L. Deneux, C. Mathiot, H. Brailly, F. Montero, I. Joyeux, P. Pouillart, W. H. Fridman

**Affiliations:** Laboratoire d'Immunologie Clinique, Institut Curie, Paris, France.

## Abstract

Interleukin 6 and C-reactive protein (CRP) were determined prior to IL-2 therapy in sera from metastatic melanoma patients. Patients with elevated serum IL-6 (> 20 pg ml-1) and/or CRP (> 10 mg l-1) levels were associated with resistance to IL-2 therapy. A correlation between high serum IL-6 levels and a shorter median survival was also observed.


Br. J. Cancer (1994), 69, 911-913 © Macmillan Press Ltd., 1994

SHORT COMMUNICATION

### Serum interleukin 6 and C-reactive protein levels correlate with resistance to IL-2 therapy and poor survival in melanoma patients

E. Tartour[1], T. Dorval[2], V. Mosseri[3], L. Deneux[4], C. Mathiot[5], H. Brailly[6], F. Montero[6], I. Joyeux[1], P. Pouillart[2] & W.H. Fridman[1]

[1]*Laboratoire d'Immunologie Clinique and INSERM U 255,* [2]*Service de Médecine Oncologique,* [3]*Departement de Biostatistique,* [4]*Laboratoire de Biochimie and* [5]*Laboratoire d'Hématologie, Institut Curie, 26 Rue d'Ulm, 75231 Paris Cedex 05, France;* [6]*Immunotech, 13276 Marseille Cedex 9, France.*

**Summary** Interleukin 6 and C-reactive protein (CRP) were determined prior to IL-2 therapy in sera from metastatic melanoma patients. Patients with elevated serum IL-6 ($>20\ pg\ ml^{-1}$) and/or CRP ($>10\ mg\ l^{-1}$) levels were associated with resistance to IL-2 therapy. A correlation between high serum IL-6 levels and a shorter median survival was also observed.

The prognosis of patients with metastatic melanoma remains poor, with a median survival that does not exceed 6 months (Koh, 1991). Most clinical trials using interleukin 2 in metastatic melanoma have shown an average response rate of about 15-25% (Dillman *et al.*, 1993; Tartour *et al.*, 1992). Therefore, since the overall response rate is low and treatment is associated with drug toxicity, many attempts have been made to predict clinical response. Up until now, only HLA phenotype has been correlated with response to IL-2 in melanoma (Marincola *et al.*, 1992). In renal cell carcinoma, patients with detectable serum interleukin 6 (IL-6) and/or CRP $>50\ mg\ l^{-1}$ before starting IL-2 treatment have a poor response to IL-2 (Blay *et al.*, 1992). Renal cell carcinoma and melanoma share some common features, such as IL-6 secretion and expression of membrane IL-6 receptor by tumour cells and the modulation of tumour cell proliferation by IL-6 (Miki *et al.*, 1989; Lee *et al.*, 1992; Lu *et al.*, 1992). This prompted us to study serum IL-6 and CRP concentrations in melanoma patients prior to IL-2 therapy, in order to evaluate their predictive value for clinical outcome.

### Materials and methods

### *Patients*

A total of 30 patients with histologically proven metastatic melanoma, stage IV according to the American Join Commission on Cancer (AJCC) classification, were included in immunotherapy protocols with IL-2 after obtaining their written informed consent. No other anti-cancer agents were given during the 3 weeks before IL-2 therapy. Patient characteristics are shown in Table I.

According to the AJCC (Beahrs *et al.*, 1988) and studies by Markowitz *et al.* (1991) and Balch *et al.* (1983), these stage IV melanomas were divided into two prognostic groups: M1a, poor prognosis with visceral metastases; M1b, intermediate prognosis with metastases in skin, subcutaneous tissue or lymph nodes beyond the regional lymph nodes.

Disease-free interval was measured as the time between definitive treatment of the primary disease to diagnosis of relapse.

A tumour response was considered complete (CR) if all measurable disease disappeared for more than 1 month. A partial response (PR) was defined as a 50% decrease in the size of the longest perpendicular cross-sectional diameter of all lesions that lasted at least 1 month without appearance of new tumour.

### *Assays*

Serum samples were obtained from all patients during the first course of IL-2 on day 0 (before starting treatment) and were frozen at $-20°C$ until the assay.

Human IL-6 was assayed using ELISA kits purchased from Immunotech (Marseille, France).

CRP was assayed by a rate immunonephelometric technique on an Erratum protein system analyser (Automatic Beckman, Beckman Instrument).

The 95th percentiles for serum CRP and IL-6 levels in normal individuals in our laboratory were $10\ mg\ l^{-1}$ and $20\ pg\ ml^{-1}$ respectively.

### *Statistical analysis*

Data were compared using a two-tailed Fisher exact test. Survival was calculated from the start of IL-2-based therapy to the date of death. Patients who had not died were censored at the date of last follow-up. Survival parameters were estimated using the Kaplan-Meier method and compared using a log-rank test. Statistical significance was defined as $P<0.05$.

### Results

### *Pretreatment serum IL-6 and CRP in melanoma patients*

High serum IL-6 and CRP levels prior to IL-2 therapy were found in respectively 26.6% (8/30) and 46% (13/28) of metastatic melanoma patients (Figure 1a and b). Elevated serum IL-6 and CRP concentrations were equally distributed among patients treated with the various IL-2 regimens (data not shown). A trend towards a correlation between IL-6 and CRP concentrations was observed, but this was not statistically significant ($P>0.05$).

### *Correlation between pretreatment serum IL-6 and CRP concentrations and clinical response*

An association between both serum CRP and serum IL-6 levels and clinical response was observed. Only 1 out of 13 patients with CRP $>10\ mg\ l^{-1}$ responded to IL-2 therapy, whereas 6 out of 15 patients with CRP $<10\ mg\ l^{-1}$ achieved a clinical response (Figure 1a). Similarly, none of the 8 patients with elevated IL-6 levels responded to IL-2 (Figure

Correspondence: E. Tartour.
Received 27 July 1993; and in revised form 1 December 1993.

**Table I** Patient characteristics

| *Characteristics* | *No. of patients* |
|---|---|
| Age (years) | |
| Median | 46 |
| Range | 20–69 |
| Male:female | 1.5 |
| Disease-free interval (months) | |
| Median | 30 |
| Range | 0–108 |
| Metastases | |
| M1a | 24 |
| M1b | 6 |
| Schedules of IL-2 regimens (per cycle) | |
| 1. IL-2: continuous i.v. infusion of $20 \times 10^6$ IU $m^{-2}$ 24 $h^{-1}$ on days 1–5, 15–18 and 29–31 | 18 |
| 2. Cisplatin: 100 mg $m^{-2}$ 24 $h^{-1}$ on day 1; and IL-2: $18 \times 10^6$ IU $m^{-2}$ 24 $h^{-1}$ on days 4–7 and 18–22 | 5 |
| 3. As 2 plus IFN-α2a: $9 \times 10^6$ IU 24 $h^{-1}$ three times a week associated with IL-2 | 7 |
| Response | |
| Partial (PR) | 6 |
| Complete (CR) | 1 |
| Stable (SD) and progressive disease (PD) | 23 |

M1a, visceral metastases; M1b, metastases in skin, subcutaneous tissue, or lymph node beyond the regional lymph node.

**Table II** Relationship between serum IL-6 and CRP levels before IL-2 therapy and prognosis and survival

| | *Prognosis* | | | *12 month survival after* | |
|---|---|---|---|---|---|
| | *Poor* | *Intermediate* | | *onset IL-2 therapy (%)* | |
| IL-6 > 20 pg $ml^{-1}$ | 8 | 0 | | 0 | |
| IL-6 < 20 pg $ml^{-1}$ | 16 | 6 | $P^* = 0.15$ | 22.5 | $P^{**} = 0.01$ |
| CRP > 10 mg $l^{-1}$ | 10 | 3 | | 13 | |
| CRP < 10 mg $l^{-1}$ | 12 | 3 | NS | 25 | $P = 0.09$ |
| IL-6 > 20 pg $ml^{-1}$ and/or CRP > 10 mg $l^{-1}$ | 13 | 3 | | 10 | |
| IL-6 < 20 pg $ml^{-1}$ and CRP < 10 mg $l^{-1}$ | 9 | 3 | NS | 30 | $P = 0.07$ |

*Fisher exact test, **log-rank test. NS, not significant.

1b). This univariate analysis was unable to predict clinical response with statistical significance, probably because of the small number of patients. However, when serum IL-6 and CRP levels were combined, a clear correlation between these markers and clinical response was demonstrated ($P = 0.02$) (Figure 1c). In fact, only one clinical response was observed in the group of 16 melanoma patients with IL-6 > 20 pg $ml^{-1}$ and/or CRP > 10 mg $l^{-1}$. In contrast, 50% of clinical responses were recorded in the group of 12 patients with normal serum IL-6 and CRP values (Figure 1c).

*Correlation between prognostic factors and serum IL-6 and CRP concentrations*

According to previous studies (Balch *et al.*, 1983; Markowitz *et al.*, 1991), these stage IV melanoma patients were divided into two prognostic groups: poor and intermediate prognosis. Although all patients with serum IL-6 levels > 20 pg $ml^{-1}$ belonged to the poor prognosis group, no clear correlation was demonstrated between serum IL-6 and CRP concentrations and the clinically defined prognostic groups (Table II). Finally, survival analysis revealed a clear correlation between a high serum IL-6 level and fatal outcome (Table II). In fact, patients with high serum IL-6 levels had a median survival of 3 months compared with 9 months in patients with low serum IL-6 levels ($P = 0.01$).

### Discussion

This study identified a group of melanoma patients with elevated serum IL-6 and/or CRP levels, which were associated with a poor clinical response to IL-2 therapy. In contrast, a 50% clinical response rate was observed in patients with normal serum IL-6 and CRP levels, whereas a clinical response of about 22% was recorded in the overall population of melanoma patients treated. No response to IL-2 therapy was recorded in patients with elevated serum IL-6 levels. The type of IL-2 regimen did not seem to influence the value of IL-6 and CRP as prognostic factors, as the clinical response was not significantly different between the various IL-2 regimens and the distribution of serum IL-6 and CRP concentrations seemed to be homogeneous between

**Figure 1** Relationship between serum CRP and IL-6 levels before IL-2 therapy and clinical response. Responders (■) and non-responders (□) to IL-2 therapy were compared with regard to normal (< 10 mg $ml^{-1}$) or pathological (> 10 mg $l^{-1}$) serum CRP (**a**), normal (< 20 pg $ml^{-1}$) or elevated (> 20 pg $ml^{-1}$) serum IL-6 concentration (**b**), and to combined data, i.e. normal serum CRP and IL-6 levels *vs* elevated serum IL-6 and/or CRP levels (**c**). Statistical analyses were performed with the Fisher exact test.

patients treated with these different protocols (data not shown).

These results are in accordance with previous studies indicating a correlation between serum CRP and IL-6 levels and clinical response to IL-2 therapy in patients with renal cell carcinoma (Blay *et al.*, 1992) and colorectal carcinoma (Broom *et al.*, 1992). Blay *et al.* found a good correlation between serum IL-6 and CRP levels which was less marked in this study.

We then wondered whether this poor IL-2 responder group corresponded to clinical prognostic groups. Patients with elevated serum IL-6 and/or CRP levels were not over-represented in the poor prognosis group with statistical significance. No relationship between serum IL-6 or CRP levels and disease-free interval, site or number of metastases was demonstrated (data not shown).

Patients with elevated serum IL-6 levels had a shorter survival than patients with low serum IL-6 levels. This is similar to the results reported by Blay *et al.* (1992), who found an association between elevated serum IL-6 levels and decreased survival in renal cell carcinoma.

The action of IL-6 on the immune system is complex, and both beneficial and adverse effects have been reported. IL-6 enhances the cytotoxic activity of NK cells (Luger *et al.*, 1989), and an anti-tumour effect of recombinant IL-6 has been reported in mice (Mule *et al.*, 1990). When transplanted in mice, a lung adenocarcinoma transfected with a cDNA coding for IL-6 lost its tumorigenicity and induced an effective immune response (Porgador *et al.*, 1992). On the other hand, high IL-6 concentrations inhibit T-cell proliferative responses and tumour necrosis factor α (TNF-α) synthesis (Aderka *et al.*, 1989; Zhou *et al.*, 1991).

Lu *et al.* (1992) showed that the growth of melanoma cells obtained from early-stage (metastically incompetent) primary lesions is inhibited by IL-6. This growth-inhibitory effect was lost in the more advanced stage (metastatically competent) derived cell lines, which also exhibited an increase in resistance to other inhibitory factors such as IL-1β, TNF-α and transforming growth factor β (TGF-β) (Lu *et al.*, 1992). These resistance phenomena were often associated with spontaneous IL-6 secretion by these advanced-stage cell lines (Lu *et al.*, 1993). Therefore, this multicytokine resistance phenotype may explain the failure of IL-2 therapy in such patients, if IL-2 acts by inducing selective cytokines or inhibitory factors.

In conclusion, our study suggests that high serum IL-6 and/or CRP levels could constitute a prognostic factor to stratify IL-2-treated melanoma patients.

This work was supported by grants from Comité de Paris de la Ligue Nationale de Lutte contre le Cancer (France) and the EC (No. B102.CT92-0316), by Institut Curie and by INSERM.

**Abbreviations:** CRP, C-reactive protein; IL-6, interleukin-6; IL-2, interleukin-2; PR, partial resonse; CR, complete response; SD, stable disease; PD, progressive disease.

### References

ADERKA, D., LE, J. & VILCEK, J. (1989). IL-6 inhibits lipopolysaccharide-induced tumor necrosis factor production in cultured human monocytes, U937 cells, and in mice. *J. Immunol.*, **143**, 3517–3524.

BALCH, C.M., SOONG, S.J., MURAD, T.M., SMITH, J.W., MADDOX, W.A. & DURANT, J.R. (1983). A multifactorial analysis of melanoma. IV. Prognostic factors in 200 melanoma patients with distant metastases (stage III). *J. Clin. Oncol.*, **1**, 126–134.

BEAHRS, O.H., HENSON, D.E., HUTTER, R.V.P. & MYERS, M.H. (1988). Melanoma of the skin (excluding eyelid). In *Manual for Staging of Cancer*, 3rd edn., pp. 139–144. J.B. Lippincott: Philadelphia.

BLAY, J.Y., NEGRIER, S., COMBARET, V., ATTALI, S., GOILLOT, E., MERROUCHE, Y., MERCATELLO, A., RAVAULT, A., TOURANI, J.M., MOSKOVTCHENKO, J.F., PHILIP, T. & FAVROT, M. (1992). Serum level of Interleukin 6 as a prognosis factor in metastatic renal cell carcinoma. *Cancer Res.*, **52**, 3317–3322.

BROOM, J., HEYS, S.D., WHITING, P.H., PARK, K.G.M., STACHAN, A., ROTHNIE, I., FRANKS, C.R. & EREMIN, O. (1992). Interleukin 2 therapy in cancer: identification of responders. *Br. J. Cancer*, **66**, 1185–1187.

DILLMAN, R.O., CHURCH, C., OLDHAM, R.K., WEST, W.H., SCHWARTZBERG, L. & BIRCH, R. (1993). Inpatient continuous-infusion interleukin-2 in 788 patients with cancer. *Cancer*, **71**, 2358–2370.

KOH, H.K. (1991). Cutaneous melanoma. *N. Engl. J. Med.*, **325**, 171–182.

LEE, J.D., SIEVERS, T.M., SKOTZKO, M., CHANDLER, C.F., MORTON, D.L., MCBRIDE, W.H. & ECONOMOU, J.S. (1992). Interleukin 6 production by human melanoma cell lines. *Lymphokine Cytokine Res.*, **11**, 161–166.

LU, C. & KERBEL, R.S. (1993). Interleukin-6 undergoes transition from paracrine growth inhibitor to autocrine stimulator during human melanoma progression. *J. Cell Biol.*, **120**, 1281–1288.

LU, C., VICKERS, M.F. & KERBEL, R.S. (1992). Interleukin 6: a fibroblast-derived growth inhibitor of human melanoma cells from early but not advanced stages of tumour progression. *Proc. Natl Acad. Sci. USA*, **89**, 9215–9219.

LUGER, T.A., KRUTMANN, J., KIRNBAUER, R., URBANSKI, A., SCHWARZ, T., KLAPPACHER, G., KOCK, A., MICKSCHE, A., MALEJCZYK, J., SCHAUER, E., MAY, L.T. & SEHGAL, P. (1989). IFN-β2/IL-6 augments the activity of human natural killer cells. *J. Immunol.*, **143**, 1206–1209.

MARINCOLA, F.M., VENZON, D., WHITE, D., RUBIN, J.T., LOTZE, M.T., SIMONIS, T.B., BALKISSOON, J., ROSENBERG, S.A. & PARKINSON, D.R. (1992). HLA association with response and toxicity in melanoma patients treated with interleukin-2-based immunotherapy. *Cancer Res.*, **52**, 6561–6566.

MARKOWITZ, J.S., COSIMI, L.A., CAREY, R.W., KANG, S., PADYK, C., SOBER, A.J. & COSIMI, A.B. (1991). Prognosis after initial recurrence of cutaneous melanoma. *Arch. Surg.*, **126**, 703–708.

MIKI, S., IWANO, M., MIKI, Y., YAMAMOTO, M., TANG, B., YOKOKAWA, K., SONODA, T., HIRANO, T. & KISHIMOTO, T. (1989). IL-6 functions as an autocrine growth factor in renal cell carcinomas. *FEBS Lett.*, **250**, 607–610.

MULE, J.J., MCINTOSH, J.K., JABLONS, D.M. & ROSENBERG, S.A. (1990). Antitumor activity of recombinant interleukin 6 in mice. *J. Exp. Med.*, **171**, 629–636.

PORGADOR, A., TZEHOVAL, E., KATZ, A., VADAI, E., REVEL, M., FELDMAN, M. & EISENBACH, L. (1992). Interleukin 6 gene transfection into Lewis lung carcinoma tumour cells suppresses the malignant phenotype and confers immunotherapeutic competence against parental metastatic cells. *Cancer Res.*, **52**, 3679–3686.

TARTOUR, E., MATHIOT, C. & FRIDMAN, W.H. (1992). Current status of interleukin-2 therapy in cancer. *Biomed. Pharmacother.*, **46**, 1–12.

ZHOU, D., MUNSTER, A. & WINCHURCH, R.A. (1991). Pathologic concentrations of interleukin 6 inhibit T cell responses via induction of activation of TGF-β. *FASEB. J.*, **5**, 2582–2585.